# Double Posterior Cruciate Ligament Sign

**DOI:** 10.3390/reports9020120

**Published:** 2026-04-14

**Authors:** Christos Koutserimpas, Charalampos Matzaroglou, Konstantinos Kaliarntas, Evdokia Billis, Nikolaos-Achilleas Arkoudis, George Triantafyllou, Maria Piagkou, John Gliatis

**Affiliations:** 1 School of Health Rehabilitation Sciences, University of Patras, 26504 Patras, Greece; matzaroglou@upatras.gr (C.M.); kaliarntas@upatras.gr (K.K.); billis@upatras.gr (E.B.); 2 Research Unit of Radiology and Medical Imaging, National and Kapodistrian University of Athens, 11528 Athens, Greece; nick.arkoudis@gmail.com; 3 Second Department of Radiology, General University Hospital “Attikon”, National and Kapodistrian University of Athens, Chaidari, 12462 Athens, Greece; 4 Department of Anatomy, School of Medicine, Faculty of Health Sciences, National and Kapodistrian University of Athens, 11527 Athens, Greece; georgerose406@gmail.com (G.T.); mapian@med.uoa.gr (M.P.); 5 Department of Orthopaedics, School of Medicine, University of Patras, 26504 Patra, Greece; gliatis@hotmail.com

**Keywords:** knee arthroscopy, meniscus, bucket handle, MRI knee, knee imaging

## Abstract

A 34-year-old male presented with persistent medial knee pain and mechanical symptoms three months after a rotational injury, with limited knee extension on examination. Magnetic resonance imaging demonstrated the double posterior cruciate ligament (PCL) sign, produced by a displaced bucket-handle tear of the medial meniscus with the fragment lying anterior and parallel to the intact PCL within the intercondylar notch. Coronal sequences confirmed displacement and loss of normal meniscal configuration. Arthroscopy verified the diagnosis, and arthroscopic partial meniscectomy was performed due to chronic displacement and poor healing potential. Following structured rehabilitation, the patient returned to full athletic activity without symptoms at one-year follow-up. This case underscores the importance of recognizing the double PCL sign as a highly specific MRI finding enabling prompt diagnosis and appropriate management of displaced bucket-handle meniscal tears, while also highlighting its radiologic–arthroscopic correlation and the clinical implications of delayed presentation on treatment strategy, and provides a clear illustrative example of this classic imaging sign for educational purposes.

**Figure 1:**
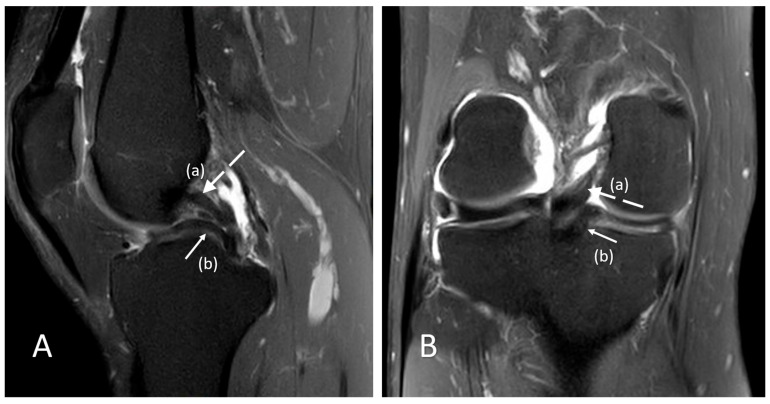
A 34-year-old man with a body mass index (BMI) = 24.6 kg/m^2^ sustained a rotational in-jury of the left knee during athletic activity (basketball practice) and subsequently developed persistent medial joint-line pain. Three months later, he continued to report mechanical symptoms with a limitation of knee extension of approximately 7°. Clinical examination demonstrated medial joint-line tenderness, while both the McMurray and Thessaly tests were positive, raising suspicion of a meniscal injury. MRI was performed on a 1.5 T scanner using a sagittal (**A**) and coronal (**B**) proton density fat-suppressed (PD-FS) sequence of the knee. Sagittal PD-FS images (Panel (**A**)) demonstrate a low-signal, curvilinear structure within the intercondylar notch running parallel and immediately anterior to the posterior cruciate ligament (PCL) [white dashed arrow (a)], producing the classic double PCL sign. This structure corresponds to a displaced bucket-handle fragment of the medial meniscus [white arrow (b)]. Coronal PD-FS images [Panel (**B**): white dashed arrow (a): PCL, (b): displaced medial meniscus fragment] confirm displacement of the torn medial meniscal fragment into the intercondylar notch [white arrow (b)] with associated loss of the normal medial meniscal body configuration. These findings are characteristic of a displaced bucket-handle tear of the medial meniscus, explaining the patient’s persistent mechanical symptoms, and arthroscopic surgical treatment was subsequently proposed. Although the double PCL sign is a well-recognized MRI finding, its recognition remains clinically important, as it represents a highly specific, though not pathognomonic, indicator of a displaced bucket-handle meniscal tear requiring timely surgical management. The sign corresponds to a displaced meniscal fragment within the intercondylar notch lying anterior and parallel to the intact PCL, creating the characteristic appearance [1,2,3,4,5]. Potential diagnostic pitfalls include the meniscofemoral ligaments (ligaments of Humphrey and Wrisberg), discoid medial meniscus, mucoid degeneration of the PCL (“tram-track” appearance), and other intercondylar structures; therefore, it should be interpreted in the appropriate clinical and imaging context [6,7,8,9].

**Figure 2:**
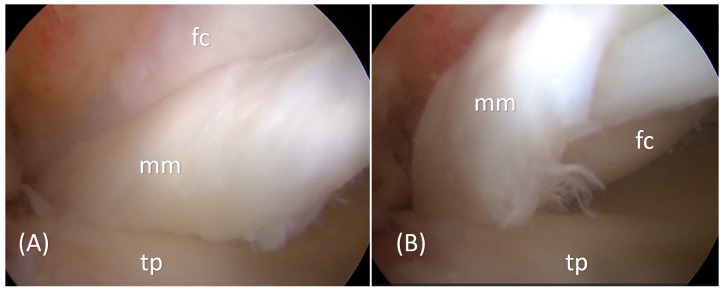
Arthroscopic evaluation of the left knee confirmed a displaced bucket-handle tear of the medial meniscus. Intraoperative views (Panels (**A**,**B**); mm: medial meniscus, tp: tibial plateau, fc: femoral condyle) demonstrate the displaced meniscal fragment folded into the intercondylar notch, causing mechanical obstruction and corresponding to the double PCL sign observed on preoperative MRI. The arthroscopic findings directly correlate with the preoperative MRI appearance, with the displaced meniscal fragment visualized intraoperatively in the same anatomical relationship to the intact PCL as identified on imaging, thereby confirming the radiologic diagnosis. The torn fragment was unstable, located predominantly within the red–white zone, and demonstrated chronic displacement, rendering meniscal repair less favorable due to limited healing potential. In addition, the fragment showed deformation and poor tissue quality, and was not adequately reducible to its native position, further limiting the feasibility of repair. Therefore, arthroscopic partial meniscectomy was performed to remove the displaced fragment and restore joint mechanics. Postoperatively, the patient followed a standard rehabilitation protocol without case-specific modifications and returned to full athletic activity within 12 weeks. At one-year follow-up, the patient reported complete resolution of symptoms, had returned to full athletic activity without limitations, and clinical examination demonstrated a painless knee with full range of motion (0–140°), absence of joint-line tenderness, and negative McMurray and Thessaly tests, indicating an excellent functional outcome. Meniscal preservation is generally preferred whenever feasible, given its role in load distribution, joint stability, and cartilage protection [10]. Current trends increasingly favor meniscal repair, even in complex tear patterns, supported by evidence demonstrating lower rates of progression to advanced osteoarthritis and subsequent knee arthroplasty compared to partial meniscectomy [11]. However, healing potential is closely related to vascular supply, which is largely confined to the peripheral red–red zone, while the inner regions demonstrate reduced vascularity [12]. In the present case, the tear was located predominantly within the red–white zone and showed chronic displacement and deformation, likely related to delayed presentation, reducing the likelihood of successful repair [13]. Therefore, partial meniscectomy was considered the most appropriate option to relieve mechanical symptoms and restore knee function. Of course, given the single-case nature of this report, the clinical outcome should be interpreted in the appropriate context. Although this approach is associated with recognized long-term risks, careful and limited resection remains an accepted treatment strategy when repair is not feasible [13,14,15].

## Data Availability

The original data presented in the study are included in the article, further inquiries can be directed to the corresponding author.
